# Associative gene networks reveal novel candidates important for ADHD and dyslexia comorbidity

**DOI:** 10.1186/s12920-023-01502-1

**Published:** 2023-09-04

**Authors:** HE Hongyao, JI Chun, Gao Xiaoyan, Liu Fangfang, Zhang Jing, Zhong Lin, Zuo Pengxiang, Li Zengchun

**Affiliations:** Medical College of Shihezi University, Shihezi, China

**Keywords:** Comorbidity, ADHD, Dyslexia, ANDSystem, Gene prioritization, Biological processes

## Abstract

**Background:**

Attention deficit hyperactivity disorder (ADHD) is commonly associated with developmental dyslexia (DD), which are both prevalent and complicated pediatric neurodevelopmental disorders that have a significant influence on children’s learning and development. Clinically, the comorbidity incidence of DD and ADHD is between 25 and 48%. Children with DD and ADHD may have more severe cognitive deficiencies, a poorer level of schooling, and a higher risk of social and emotional management disorders. Furthermore, patients with this comorbidity are frequently treated for a single condition in clinical settings, and the therapeutic outcome is poor. The development of effective treatment approaches against these diseases is complicated by their comorbidity features. This is often a major problem in diagnosis and treatment. In this study, we developed bioinformatical methodology for the analysis of the comorbidity of these two diseases. As such, the search for candidate genes related to the comorbid conditions of ADHD and DD can help in elucidating the molecular mechanisms underlying the comorbid condition, and can also be useful for genotyping and identifying new drug targets.

**Results:**

Using the ANDSystem tool, the reconstruction and analysis of gene networks associated with ADHD and dyslexia was carried out. The gene network of ADHD included 599 genes/proteins and 148,978 interactions, while that of dyslexia included 167 genes/proteins and 27,083 interactions. When the ANDSystem and GeneCards data were combined, a total of 213 genes/proteins for ADHD and dyslexia were found. An approach for ranking genes implicated in the comorbid condition of the two diseases was proposed. The approach is based on ten criteria for ranking genes by their importance, including relevance scores of association between disease and genes, standard methods of gene prioritization, as well as original criteria that take into account the characteristics of an associative gene network and the presence of known polymorphisms in the analyzed genes. Among the top 20 genes with the highest priority DRD2, DRD4, CNTNAP2 and GRIN2B are mentioned in the literature as directly linked with the comorbidity of ADHD and dyslexia. According to the proposed approach, the genes OPRM1, CHRNA4 and SNCA had the highest priority in the development of comorbidity of these two diseases. Additionally, it was revealed that the most relevant genes are involved in biological processes related to signal transduction, positive regulation of transcription from RNA polymerase II promoters, chemical synaptic transmission, response to drugs, ion transmembrane transport, nervous system development, cell adhesion, and neuron migration.

**Conclusions:**

The application of methods of reconstruction and analysis of gene networks is a powerful tool for studying the molecular mechanisms of comorbid conditions. The method put forth to rank genes by their importance for the comorbid condition of ADHD and dyslexia was employed to predict genes that play key roles in the development of the comorbid condition. The results can be utilized to plan experiments for the identification of novel candidate genes and search for novel pharmacological targets.

**Supplementary Information:**

The online version contains supplementary material available at 10.1186/s12920-023-01502-1.

## Background

Attention deficit hyperactivity disorder (ADHD) and developmental dyslexia (DD) are both prevalent and complicated pediatric neurodevelopmental disorders that have a significant influence on children’s learning and development. More than 80% of children with ADHD and 60% of children with dyslexia fulfill at least one problem diagnostic criterion [1, 2]. ADHD is diagnosed individuals who exhibit inattention, hyperactivity, and impulsive behavior problems, with a prevalence of about 7.2% according to a recent meta-analysis [3]. The prognosis of ADHD is poor, with some children retaining symptoms into adulthood, and a study found that the risk of premature death is twice as high in those affected with ADHD, bringing a heavy burden to society and families [4]. Learning challenges are also a major motivator for ADHD patients to seek medical attention. ADHD is most commonly connected with DD among learning disorders [5]. Clinically, the comorbidity incidence of DD and ADHD is as high as 25–48% [6]. DD is a condition in which an individual with a normal IQ and educational opportunities, without hearing or nervous system problems, has much lower reading ability than the equivalent age level [7]. In nations that use alphabets, the identification rate of phonetic characters varies between 5 and 17.5% [8, 9]. In nations that use ideograms, such as Chinese characters, the detection rate of minors ranges from 3 to 16% [10, 11]. Dyslexia, as a chronic handicap, not only impedes children’s language acquisition, but also has an impact on arithmetic and other subjects. The reduction of academic performance may result in poorer self-esteem and increased loneliness in children, leading to psychological disorders such as anxiety, depression, and fatigue [12].

According to several studies, comorbid dyslexia may be a sign of ADHD in a subset of affected children who have more severe cognitive impairments and impaired executive and non-executive functioning [13]. Children with comorbidities exhibited greater secondary difficulties, such as low self-esteem, behavioral problems, and higher school dropout rates, than children with ADHD or dyslexia alone [14]. Furthermore, those with comorbid dyslexia and ADHD are more likely to develop additional debilitating diseases, and the prevalence of comorbid symptoms increases with age [15].

Both ADHD and dyslexia are related to impaired brain function [16]. They may share common abnormal changes in brain structure and functional characteristics, resulting in common cognitive impairment, which may be affected by common genetic factors [17]. The pathological etiology of ADHD and dyslexia is not fully understood, but genetic factors play a significant role in both diseases. It has been reported that children with a family history of ADHD or dyslexia were at a higher risk [18]. According to twin studies, the heritability of ADHD is around 0.74 [19], whereas the heritability of dyslexia is 0.4 ~ 0.6 [20].

Candidate genes for ADHD are mostly related to dopamine, 5-hydroxytryptophane, norepinephrine, and other neurotransmitter systems [21]. Several GWAS studies on ADHD have identified genes involved in processes important for brain development, such as cell adhesion and synapse formation [22]. DCDC2, DYX1C1, and KIAA0319 may be pathogenic genes that contribute to dyslexia [23]. According to linkage studies, multiple chromosomal regions have been linked to ADHD and dyslexia, including 6q12-q14, 15q, 16p, 5p, and 17p [24]. The most researched chromosomal region is 6p21-22, which contains significant genes including DCDC2 and KIAA0319 [25]. The DCDC2 gene is now known to be linked to dyslexia. According to a recent meta-analysis, the C allele of the rs807701 polymorphism of the DCDC2 gene can increase the risk of dyslexia [26]. Couto et al. investigated the link between the DCDC2 gene and ADHD. Their findings revealed a robust link between attention deficit, hyperactivity, and impulsive symptoms [27]. DCDC2 may influence ADHD symptoms via gene-gene interaction with KIAA3019, as well as gene-environment interactions with factors related to socioeconomic status [28]. Furthermore, functional impairment of the norepinephrine system plays a significant role in the pathogenic mechanism of ADHD and dyslexia [29], with the ADRA2A gene being related to ADHD in a number of studies, as well as ADHD with comorbid dyslexia [30]. Other frequent susceptibility genes for ADHD and dyslexia include DYX1C1 and DRD4.

Nowadays, there are large amounts of data on these diseases, allowing the construction of associative gene networks that describe the potential molecular mechanisms of interactions between the diseases. There are a number of resources in that allow the reconstruction of such associative gene networks, such as MetaCore [31], Ingenuity [32], and ANDSystem [33, 34]. A number of studies were performed using the ANDSystem tool, including an analysis of proteomic data on *Helicobacter pylori* infection [35], an analysis of the effect of tissue-specific gene knockouts and the search for potential drug targets [36], analysis of gene networks related to the life cycle of hepatitis C virus [37], as well as analyses of the comorbidity of bronchial asthma and tuberculosis [38], preeclampsia, diabetes, obesity and glaucoma [39], or of asthma and hypertension [40].

The purpose of this study was to prioritize potential genes based on gene network reconstruction and analysis explaining the connection between ADHD and dyslexia. The relationship between the two is detailed in depth in this research via the association network reconstructed using the ANDSystem tool, and the comorbidity-related genes were sourced from the GeneCards web server. Standard prioritizing approaches (Endeavour and ToppGene) were used, as well as novel methods that took into consideration the topology of the ADHD/dyslexia gene network and the correlation of gene polymorphisms with disease risk. The predicted genes can be used to plan genotyping experiments in order to find genes that predict a susceptibility for dyslexia and ADHD comorbidity.

## Methodology

The reconstruction of associative gene networks related to ADHD and dyslexia was carried out using the ANDSystem tool [33, 34], which can automatically analyze scientific publications to extract data on the molecular genetic interactions and associations of proteins, genes, metabolites, drugs, and microRNAs with diseases, biological processes, drug side effects, or the phenotypes of various organisms. The main modules of ANDSystem are knowledge extraction module, ANDCell knowledge base and user interface ANDVision. The knowledge extraction module is based on text mining technology, using object name dictionary and semantic template. The compilation of the dictionary is based on the automatic extraction of the names and synonyms of biological objects from external databases and scientific publications. Semantic templates are structured records that list object types, dictionaries, regular expressions for text analysis, and interactive semantic descriptions. The ANDSystem knowledge base was built on the basis of a large-scale analysis of over 25 million abstracts of scientific papers listed in the PubMed database. Information on molecular genetic interactions from different factual databases, such as IntAct, MINT, and others, was integrated into ANDSystem. In total, more than seven million facts regarding molecular genetic interactions and associations are available in the ANDSystem knowledge base. In the current study, we used ANDSystem version 2021 [41], which is based on the analysis of all PubMed abstracts up to 2021, as well as information obtained from external databases that were available in 2021. No custom changes have been made in present study. As the ANDVisio allows to analyze the molecular-genetic networks it was applied to find the node connectivity and betweenness centrality coefficients of nodes in the hypoglycemia gene network. These parameters were calculated with function “Statistics” of the “Analysis” section of ANDVisio. The cross-talk specificity (CTS) values were calculated by ANDVisio function “Intelligent Filtration.” CTS was calculated according to the formula: CTS = Ki/Mi, where Ki is a number of links that the i gene has in the analyzed gene network; Mi is a number of links that the i gene has in the global human gene network of ANDSystem .

Enriched gene ontology (GO) biological processes were identified using the DAVID service [42], with default settings. To evaluate the centrality of vertices in the graphs of gene networks, Cytoscape was used to calculate the degree centrality (DC), total centrality (CC), and betweenness centrality (BC) [43].

**Fig. 1:**
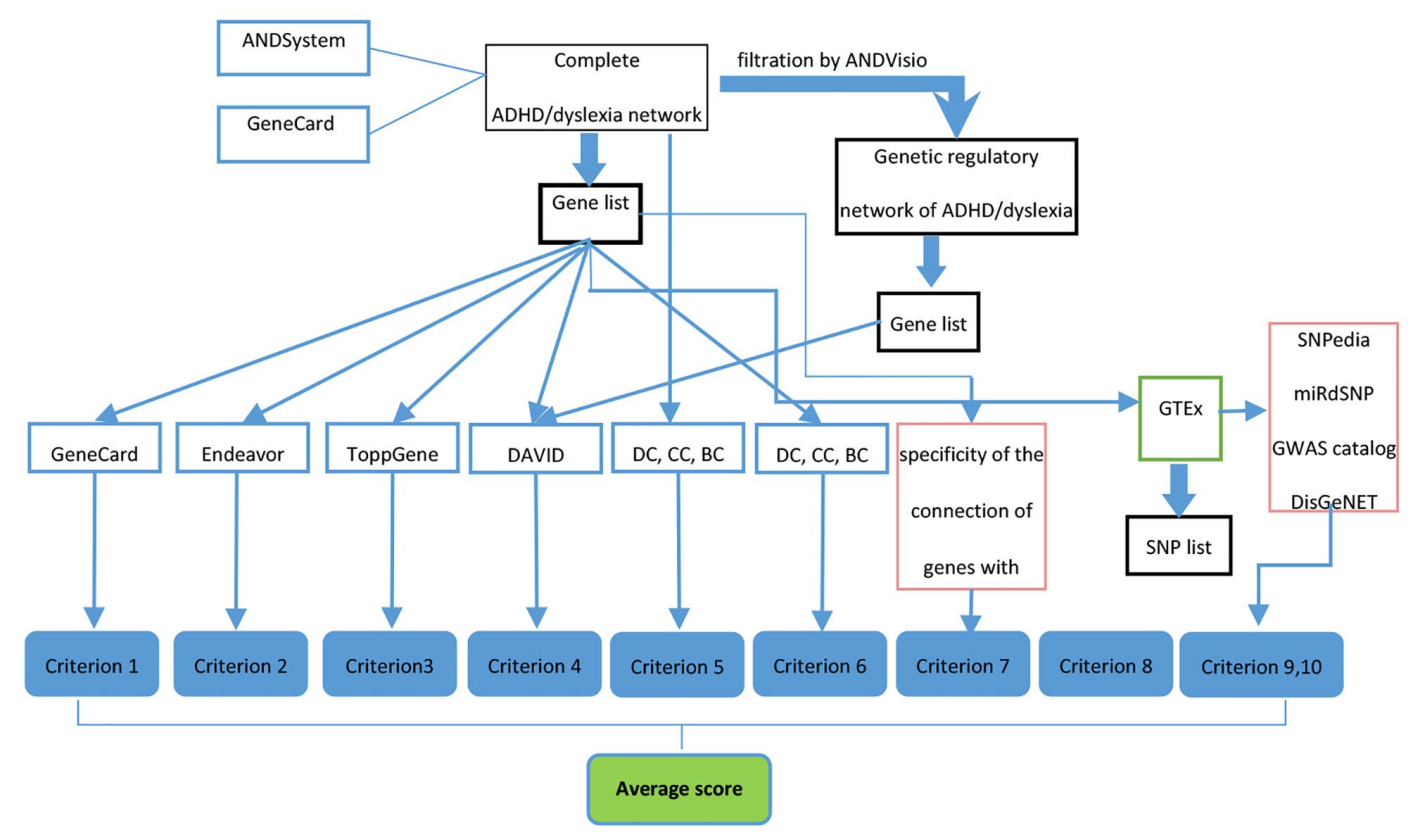
A general scheme for calculating criteria of gene

A schematic illustration of the gene prioritization algorithm that includes 10 criteria is shown in Fig. 1.

### Criterion 1

calculated using the GeneCards for gene prioritization, (https://www.genecards.org/) [44, 45]: Rank1_i_ = Rank(X_i_), where X is a sorted list of genes according to average of the relevance score rank. GeneCards is a comprehensive human gene database that provides brief information on all known and anticipated human genes, proteins, transcription, genetics, and functions. The link between genes and illnesses is also included in the information function of GeneCards, and the relevance score of the link between genes and diseases may be calculated. In this case, genes are sequenced based on their relevance score.

### Criterion 2

calculated using the Endeavour system for gene prioritization, (https://endeavour.esat.kuleuven.be/Endeavour.aspx) [46, 47]: Rank2_i_ = Rank(X_i_), where X is a sorted list of genes according to Endeavour output, and i is the gene number. The algorithm was used with default settings, and the list of genes from the complete ADHD/dyslexia network was utilized as the input for the test and training sets.

### Criterion 3

calculated with the gene prioritization system, ToppGene (https://toppgene.cchmc.org/prioritization.jsp) [48, 49]: Rank3_i_ = Rank(X_i_), where X is a sorted list of genes according to ToppGene output, and i is the gene number. The algorithm was used with default settings. The genes from the complete ADHD/dyslexia network were entered as the input, and the list of genes from the complete ADHD/dyslexia network, from which the analyzed genes were excluded, was provided as a training set.

### Criterion 4

GO biological processes enriched in the complete and genetic regulatory networks of ADHD/dyslexia. This score was computed as Rank4_i_ = Rank(X_i_), where X is a sorted list of genes according to N_i_=N1_i_ + N2_i_, where N1_i_ is the total number of enriched GO biological processes in complete networks of ADHD/dyslexia in which gene i was involved, N2 was calculated the same as N1 for genetic regulatory networks of ADHD/dyslexia (see Additional file 1: Table S2).

### Criterion 5

calculated for gene i as Rank5_i_ = Rank(X_i_), where X is a sorted list of genes according to average measure of the value of DC, CC, and BC for each gene from the complete network of ADHD/dyslexia.

### Criterion 6

calculated in the same way as Criterion 5 using the genetic regulatory network of ADHD/ dyslexia instead of the complete network of ADHD/ dyslexia.

### Criterion 7

Rank7_i_ = Rank(X_i_), where X is a sorted list of genes according to specificity of the connection of genes with biological processes associated with ADHD and dyslexia. To arrive at this score, at the first step, a list of biological processes connected with ADHD and dyslexia according to ANDSystem was constructed. In ANDSystem, the biological processes are named “Pathways”. They were filtered manually or using a script to leave only the Gene Ontology biological processes. Then the list of Gene Ontology biological processes associated with ADHD was intersected with the list of Gene Ontology biological processes associated with dyslexia. This intersection constitutes the biological processes connected simultaneously with ADHD and dyslexia. Then, all the interactions between all human genes (proteins) and all Gene Ontology biological processes were downloaded from the Gene Ontology server (http://current.geneontology.org/products/pages/downloads.html). Furthermore, we established the interactions between studied genes and biological processes using the downloaded files. We then divided all the Gene Ontology biological processes into 2 groups. The first group (the test set) contained the biological processes connected simultaneously with both studied diseases from step 1. The second group (control set) encompassed all of the remaining GO-based biological processes. The specificity of the connection between genes/proteins and the test set of Gene Ontology biological processes was then evaluated by applying Student’s *t*-test using the function stats.ttest_ind with the parameter equal_var = false, using the scipy.stats package in Python [50, 51]. An FDR correction for multiple comparisons was conducted using the p.adjust function (Y, “FDR”) of the “stats” package in the programming language R.

### Criterion 8

Rank8_i_ = 1 if SNPs from list Y was present in gene i, otherwise Rank8_i_ was equal to the maximal rank for list X (Rank8_i_ = 231), since the presence of such polymorphisms is of great importance for genotyping. List Y included all SNPs for each gene from X that were found in the eQTL gene region with a frequency of the minor allele in at least 5% of the sequences in the 1000Genomes database. A threshold of 5% allows the detection of MAF polymorphisms with a high degree of probability using available genotyping arrays, and it is often used in genomic analysis [52, 53]. To calculate this score, the GTEx resource (http://www.gtexportal.org) [54] was consulted. It provides information on the variability of global expression of genes and SNPs affecting the level of gene expression. For the analyzed genes, all SNPs localized in the region of the eQTL were taken from the database. Such SNPs may be relevant to the development of diseases [55, 56]. Then, only the SNPs that alter the expression of the analyzed genes in the brain were selected. As the next step, the prevalence of the minor alleles among the sequences in the 1000Genomes database was estimated for SNPs in the eQTL region. The analysis was carried out using the NCBI database (https://www.ncbi.nlm.nih.gov/omim/) [57]. For further analysis, only SNPs that had a minor allele frequency of at least 5% among the sequences in the 1000Genomes database were selected (for most of the found SNPs, the minor allele frequency was 20% or higher).

### Criterion 9

Rank9_i_ = 1 if any gene i has an SNP associated with either ADHD or dyslexia that is present in list Y, otherwise Rank8_i_ was equal to 231.

### Criterion 10

Rank10_i_ = 1 if in list Y for gene i an SNP is associated with some disease comorbid with ADHD or dyslexia was present, otherwise Rank9_i_ was equal to 231. Manual analysis of PubMed publications was conducted to generate a list of diseases comorbid with ADHD and dyslexia. For ADHD, we manually examined 594 PubMed publications found by the query, “ADHD comorbid diseases”, and filtered via the parameter, “Free full text”. For dyslexia, 422 PubMed publications obtained with the query “dyslexia comorbid diseases”, and filtered by the parameter, “Free full text”, were analyzed. For each gene, the final score was computed as the average value of ranks formulated according to criteria 1–10.

## Results and discussion

### Associative gene networks related to ADHD and dyslexia

In order to identify potential molecular genetic mechanisms underlying the development of ADHD and dyslexia, we compiled a list of 599 genes/proteins associated with ADHD and 167 genes/proteins associated with dyslexia according to ANDSystem (Additional file 1: Table S1). The gene network of ADHD included 148,978 interactions between 321 genes and 278 proteins, including 623 activity regulations, 16 degradation regulations, 863 expression regulations, 4931 transport regulations, 4838 downregulations, 131,844 associative interactions, and 5863 protein-protein interactions. In the ANDSystem, associative interaction is a special category of interactions reflecting any type of relations between two objects listed above.

The gene network of dyslexia included 27,083 interactions between 92 genes and 75 proteins, including 31 activity regulations, 1 degradation regulations, 48 expression regulations, 266 transport regulations, 381 downregulations, 26,023 associative interactions, and 333 protein-protein interactions. In order to complement the gene set of the molecular genetic mechanisms underlying the development of ADHD and dyslexia, we compiled a list of 1842 genes associated with ADHD and 921 genes associated with dyslexia according to GeneCards (Additional file 1: Table S1). There are suggestions in the literature that putative candidate genes for the development of comorbid conditions between a pair of diseases are often simultaneously associated with both diseases [58, 59]. The network of interactions between genes and proteins, associated simultaneously with ADHD and dyslexia (complete ADHD/dyslexia network), which was constructed by intersecting the ADHD and dyslexia networks, included 231 genes and 353 proteins.

The enriched GO biological processes (p-value < 0.01 with FDR correction) for genes/proteins associated with ADHD were identified using the DAVID system. The most significant GO biological processes included positive regulation of transcription from RNA polymerase II promoters, positive regulation of transcription, signal transduction, positive regulation of cell proliferation, immune response, negative regulation of transcription from RNA polymerase II promoters, inflammatory response, negative regulation of transcription, positive regulation of GTPase activity, transcription, G-protein coupled receptor signaling pathways, and chemical synaptic transmission(Additional file 1: Table S2). For genes/proteins associated with dyslexia, the most significant GO biological processes included regulation of chemical synaptic transmission, nervous system development, response to drugs, negative regulation of apoptotic process, transport, cell proliferation, intracellular signal transduction, signal transduction, positive regulation of GTPase activity, positive regulation of transcription from RNA polymerase II promoters, negative regulation of transcription, and negative regulation of transcription from RNA polymerase II promoters (Additional file 1: Table S2).

The most highly enriched GO biological processes for genes/proteins associated simultaneously with ADHD and dyslexia (complete ADHD/dyslexia network) included chemical synaptic transmission, ion transmembrane transport, response to amphetamine, neuronal migration, locomotory behavior, nervous system development, transport, cell adhesion, and signal transduction (Additional file 1: Table S2). These processes may be the most significant for the comorbid relationship between ADHD and dyslexia.

The GO biological processes that were enriched for the ADHD network, and were not featured in the list of enriched processes for the complete ADHD/dyslexia network, included G-protein coupled receptor signaling pathway, circadian regulation of gene expression, neuropeptide signaling pathway, and chemokine mediated signaling pathway. The GO biological processes that were enriched only in the dyslexia network included store-operated calcium entry, G2/M transition of the mitotic cell cycle, homophilic cell adhesion via plasma membrane adhesion molecules, potassium ion transmembrane transport, and ER to Golgi vesicle-mediated transport. Such processes appear to be more pertinent to the mechanisms of development of either ADHD or dyslexia without mutual comorbidity.

The GO biological processes that were enriched only for the complete ADHD/dyslexia network and not for the individual ADHD or dyslexia networks included chemical synaptic transmission, signal transduction, response to amphetamine, and negative regulation of neuronal apoptotic processes.

**Fig. 2:**
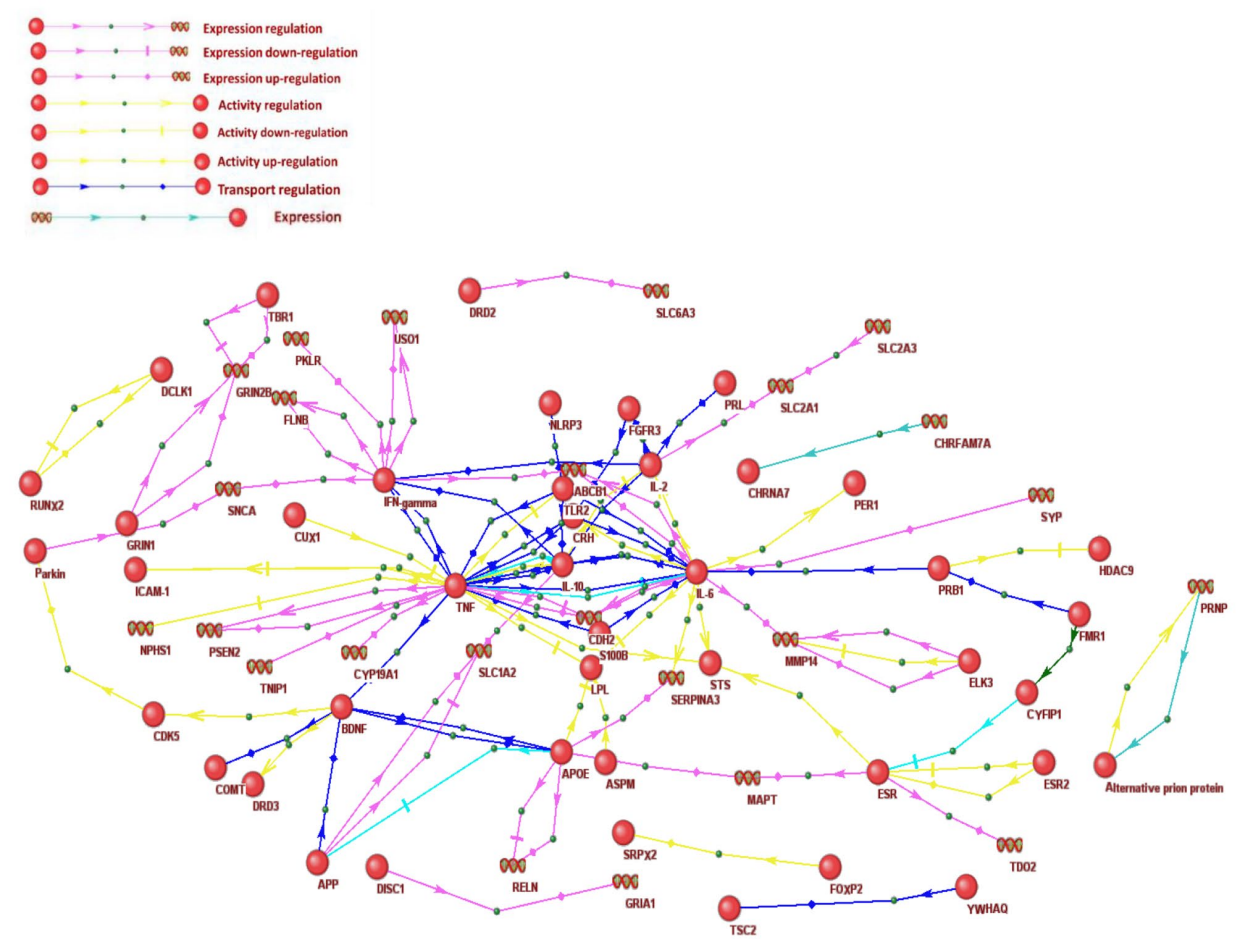
Genetic regulatory network of ADHD/dyslexia. Proteins are presented by circles and genes are represented by DNA helices. The diagram was rendered using the ANDVisio plugin ANDSystem, and gene/protein notations are given according to the ANDVisio output

It is known that genetic regulation is paramount for the genetic variability in diseases across patients [60, 61]. The genetic regulatory network of ADHD/dyslexia, including interactions between genes involved in expression and activity regulation, including up- and downregulation, as well as transport regulation, is shown in Fig. 2. As shown in Fig. 2 the general regulatory network can be divided into at least five subnetworks, including four small subnetworks containing from 2 to 3 participants (for example, DRD2 protein → SLC6A3 gene). These subnetworks appear to be unconnected with the core of the regulatory network, because in the ANDSystem classification they were connected only by associative interaction type. It was also important to evaluate the enrichment of GO biological processes for genes/proteins from the genetic regulatory network of ADHD/dyslexia (Additional file 1: Table S2). Notably, some new enriched GO biological processes were identified in this network, including G-protein coupled receptor signaling pathway, response to estradiol, circadian rhythm, positive regulation of MAPK cascade, neuropeptide signaling pathway, positive regulation of tyrosine phosphorylation of Stat3 protein, intracellular signal transduction, and G2/M transition of mitotic cell cycle. These processes were not significantly enriched within the whole ADHD/dyslexia network. The GO processes that were simultaneously significantly enriched in the complete and genetic regulatory network of ADHD/ dyslexia include the negative regulation of signal transduction, positive regulation of transcription from RNA polymerase II promoters, chemical synaptic transmission, response to drugs, ion transmembrane transport, nervous system development, cell adhesion, and neuronal migration.

One of the most central regulatory nodes for both the complete and genetic regulatory network of ADHD/dyslexia is TNF-α (Fig. 2). We observed that this gene is involved in a large number of enriched GO biological processes. For example, it participates in negative regulation of transcription from RNA polymerase II promoters, MAPK cascade, positive regulation of apoptotic processes, the inflammatory response, positive regulation of transcription, immune response, and negative regulation of transcription.

TNF neurobiological impacts include the promotion of the development, differentiation, and repair of keratinocytes and neurons [62], neuroendocrine control [63], as well as changes in the production and metabolism of numerous neurotransmitters [64]. TNF may be involved in the development of neuropsychological deficits and peripheral neuropathies, offering clues for the genetic basis of DD and ADHD comorbidity.

TNF can directly alter pain, sleep, and other behavioral activities [65], implying that TNF can influence ADHD behavior in both direct and indirect ways. TNF-induced inflammatory and immunological responses are significant in the etiology of ADHD [66]. The pro-inflammatory cytokine TNF-α is thought to play a key role in brain inflammation [67]. Increased pro-inflammatory cytokine production triggers free radical formation and leads to alterations in glutamatergic neurotransmission, resulting in an increase of neuronal excitotoxicity [68]. Additionally, pro-inflammatory cytokines play a key role in tryptophan metabolism and dopaminergic pathways that are involved in the pathogenesis of ADHD [69]. According to research, there is aberrant TNF-α expression in animal models of attention deficit, and there is a close association between the neurotrophic factor gene and brain development [70]. TNF-α can play a role in neurobiology by promoting the restoration of nerve cells and neuronal differentiation, but a lower amount will impact neurotransmitter metabolism and synthesis. High levels of prenatal testosterone can act independently across development on both the thymus and the brain [71]. Thus, hemispheric laterality, immune function [72], and the incidence of dyslexia (via a direct effect on early brain development) would all be affected [73]. Galaburda et al. performed autopsies on the brains of patients with DD and found typical neuronal abnormalities, including ectopic, smaller somatic cells in the cerebellar gyrus and sensory thalamus [74]. The link between dyslexia and immune dysfunction laid the groundwork for further research. The present study does not cover all aspects linking ADHD and DD, but rather suggests an association between ADHD and DD comorbidity via inflammatory pathways. It is interesting that the TNF gene, which has high centrality, was found to be connected in the regulatory network with BDNF, which also has a high centrality value (Fig. 2). It is known that biological networks are characterized by a low degree of assortative connectivity, i.e. vertexes with a large number of connections are rarely connected with each other. Thus, the uncovered interactions between TNF and BDNF may indicate a special role of this connection for the comorbid state of ADHD and DD. Furthermore, BDNF can suppress the expression of TNF and decrease its activity [75]. It was previously demonstrated that the activity of BDNF is reduced in both ADHD and DD [76, 77]. As shown in Fig. 2, BDNF is able to enhance the level of transport regulation of the COMT gene [78]. Additionally, the various biological processes featured in the pathogenesis of ADHD and DD, as well as their comorbid development, including chemical synaptic transmission, nervous system development and signal transduction, are connected through regulatory interactions.

### Prioritization of candidate genes

Gene prioritization is an important task in studies aimed at candidate gene identification. The available tools for gene prioritization include GeneCards, Endeavour [41, 42], ToppGene [43, 44], and DIR [79]. In GeneCards (http://www.genecards.org/), the query and correlation of illness-related genes with the rating score (relevance score) is sorted by relevance score from high to low. Endeavour, ToppGene, and DIR allow one to rank a test set of genes based on a training set of genes according to certain criteria characterizing the proximity of genes from the test set to the genes from the training set. The methods of these resources employ properties of the vertices of gene network graphs, genetic information (co-localization in the genome), functional properties of genes (involvement in the same GO categories), and other aspects. To search for candidate genes that might play an important role in the molecular genetic mechanisms of ADHD and DD comorbidity, we utilized a combination of GeneCards (criterion 1), Endeavour (criterion 2) and ToppGene (criterion 3). Additionally, to take into account the structure of the gene network, describing the interactions between ADHD and dyslexia, as well as polymorphisms in the genes associated with the studied diseases, criteria 4–10 were used. In particular, information about polymorphisms was used in criteria 8–10. All genes with known polymorphisms had a minimal rank (equal to 1), while the rank of remaining genes had the maximal value (equal to 213). This allowed us to provide criteria 8–10 with more weight compared to other criteria. We believe that the presence of polymorphisms in the studied genes is important for the development of comorbidity. The values of the listed scores for the top ten genes from the complete ADHD/dyslexia network are shown in Table 1. According to criterion 1, the top ten most important genes/proteins, sorted by the “relevance score” indicator, were DRD4, MAP1B, SLC6A3, DRD2, DRD3, CNTNAP2, DCDC2, GRIN2B, KIAA0319, and FOXP2 (Additional file 3: Table S3). According to criterion 2, the top ten most important genes/proteins, sorted by the “P-value” indicator, were GRIN1, GRIA1, GABRA1, GABRG2, GABRB1, GABRB3, GRIN2B, PSEN1, CHRNA4, and SLC1A2 (Additional file 4:Table S4). For criterion 3 the list of the top ten genes/proteins, ranked according to the “Average Score” indicator, included CDK5, GRIN2B, GRIN1, ESR1, RYR1, CHRNA7, CHRNB2, DRD4, CHRNA4, and GRIA1 (Additional file 5:Table S5). Criterion 4 suggested that for both the complete and genetic regulatory network of ADHD/dyslexia, TNF was involved in the greatest number of over-represented GO biological categories, including 122 processes (Additional file 6: Table S6). Ranking by criterion 4 demonstrated that for the 20 genes/proteins (TNF, IL6, DRD2, APOE, SNCA, CDK5, IL10, APP, TLR2, DRD1, CHRNB2, DRD3, ESR1, RELN, IFNG, ADRA2A, GRIN1, DMD, OPRM1, CASP3), the total number of GO biological processes in which these genes/proteins participated with respect to the complete and genetic regulatory network of ADHD/dyslexia was more than 70 (Fig. 2).

**Table 1:** Top 20 genes with the highest priority according to average rank

Gene name	Rank 1	Rank 2	Rank 3	Rank 4	Rank 5	Rank 6	Rank 7	Rank 8	Rank 9	Rank 10	Average rank
OPRM1	45	21	18	17	78	43	31	1	1	1	25.6
CHRNA4	29	9	9	30	48	28	5	1	231	1	39.1
SNCA	56	54	15	5	24	86	18	1	231	1	49.1
GRIN1	43	1	3	15	13	6	1	1	231	231	54.5
MAPT	67	18	30	45	16	86	71	1	231	1	56.6
DRD2	4	31	49	3	26	9	2	1	231	231	58.7
DLG4	59	13	24	26	5	5	13	1	231	231	60.8
GABRA6	120	29	55	59	53	38	40	1	231	1	62.7
GRM3	164	19	37	62	33	21	63	1	231	1	63.2
ESR1	58	22	4	12	15	13	65	1	231	231	65.2
DRD4	1	52	8	28	84	30	7	1	231	231	67.3
CACNA1A	19	15	11	36	45	86	11	1	231	231	68.6
GABBR1	182	20	51	65	39	23	81	1	231	1	69.4
CNTNAP2	6	57	84	40	17	8	23	1	231	231	69.8
SCN1A	110	50	74	49	36	86	71	1	231	1	70.9
GABRA1	49	3	17	62	30	86	22	1	231	231	73.2
DRD5	64	12	59	32	54	33	23	1	231	231	74
IL6	40	95	57	2	10	17	66	1	231	231	75
GRIN2B	8	7	2	51	3	3	9	231	231	231	77.6
PICK1	79	106	87	39	76	86	81	1	231	1	78.7

According to criteria 5 and 6, the genes/proteins with the highest centrality index for both the complete and genetic regulatory network of ADHD/dyslexia were SLC6A3, IL6, GFAP, GFAP, and DLG4. The highest centrality index for only the complete network was for genes LPL, IL6, CDH2, ESR2 and SLC1A2, and for the genetic regulatory network of ADHD/dyslexia, genes BDNF, APP, GRIN2B, COMT, DLG4 (Additional file 7: Table S7).

According to criterion 7, 80 genes/proteins are specifically associated with the test set of biological processes with a FDR corrected *p*-value < 0.05 (Additional file 8: Table S8). Among the genes most significantly associated with the test set were GRIN1, DRD2, CHRNB2, CDK5, CHRNA4, DRD3, DRD4, SLC6A3, GRIN2B, and DRD1.

**Fig. 3:**
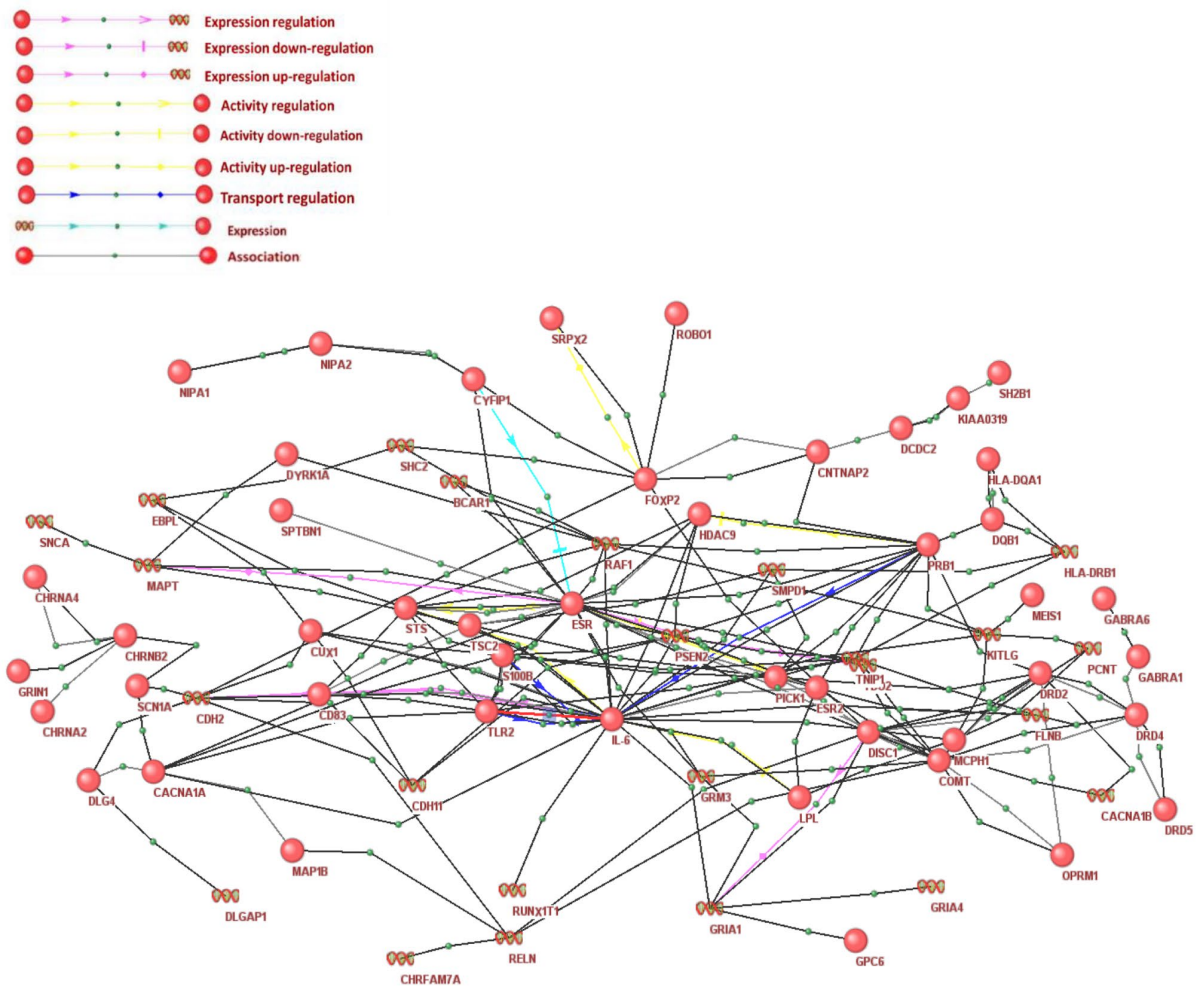
Network of interactions between genes/proteins that had SNPs determined by criteria 8–10

Criterion 8 showed that of the 231 analyzed genes, 93 had SNPs found in the eQTL database. Moreover, we revealed that there were 6170 SNPs (Additional file 9: Table S9). The highest number of SNPs (> 7 per 1000 nucleotides) was observed for the genes OPRM1, MAPT, DRD2, DCDC2, KIAA0319, and STS (Fig. 3). Among these, two polymorphisms in the OPRM1 and STS genes were associated with ADHD, while ten polymorphisms in DCDC2 and KIAA0319 were linked to dyslexia (Fig. 3). These genes had the highest priority according to criterion 9. The eight polymorphisms in genes OPRM1, CHRNA4, SNCA, PICK1, and MAPT were linked to any disease comorbid with ADHD or dyslexia (e.g., nicotine dependence, alcohol dependence, Alzheimer’s disease, general substance dependence, addiction to opioids or heroin, schizophrenia, obesity, Parkinson’s disease, or major depressive disorder). According to criterion 10, the genes OPRM1, CHRNA4, SNCA, PICK1, and MAPT had the highest priority(Additional file 10: Table S10).

After reviewing the average rank (Additional file 11: Table S11), twenty genes (OPRM1, CHRNA4, SNCA, GRIN1, MAPT, DRD2, DLG4, GABRA6, GRM3, ESR1, DRD4, CACNA1A, GABBR1, CNTNAP2, SCN1A, GABRA1, DRD5, IL6, GRIN2B, PICK1) had the highest priority (Table 1). According to the OMIM [80] and MalaCards [81] databases, all genes presented in Table 1, except for SNCA, CACNA1A, SCN1A, and GABRA1, appeared to be associated with ADHD and/or dyslexia. However, the associations of these genes with ADHD and/or dyslexia were subject to controversy in the literature^[[82, 83]]^.

The top gene OPRMI (1st) is found on chromosome 6q24-25, a genomic region linked to ADHD and dyslexia. OPRMI encodes human opioid receptor, which is a member of the G-protein-coupled receptor family and is involved in substance-related reward pathways. The opioid system may be implicated in the reward and reinforcement control abnormalities seen in ADHD, notably the defective signaling of delayed rewards caused by changes in motivational processes [84]. The same opioid receptor gene (OPRM1) polymorphism has been linked to dispositional and neural sensitivity to social rejection [85], as well as to parent-child relationships [86], implying that the opioid (reward) system is also involved in the regulation of social relations and behavior. The current findings point to the opioid system playing a particular pathophysiological role in the increased sensitivity to both behavior disruption (ADHD) and problematic drug use. However, there is no direct discussion in the literature on OPRM1 about the comorbidity of ADHD and dyslexia.

The second-ranked gene, CHRNA7, is found on human chromosome 15q13.3, a region of the genome linked to ADHD and dyslexia [87]. CHRNA7 encodes a nicotinic acetylcholine receptor. Neuro-nicotinic basic cholinergic receptors are ligand-gated ion channels found in the human cerebral cortex, hippocampus, thalamus, and striatum. They play roles in attention, memory, and cognition, with links to neurodegenerative disorders, epilepsy, nicotine dependency, ADHD, and dyslexia [88]. Dopamine and acetylcholine are both neurotransmitters that play crucial roles in the human body, and dopamine release can be enhanced by stimulating the nicotinic acetylcholine receptor [89]. Clinical studies using nicotine and nicotine agonists have shown increased performance in attention-related activities, including in individuals with ADHD [90]. In addition to the standard auditory and phonological problems, dyslexia is characterized by delayed attention transfer [91]. The nicotinic system of the brain is primarily responsible for attention shift, and some studies have shown a particular link between attention and CHRNA4 [92], which also establishes the pathogenic basis of the nicotinic receptor gene in dyslexia.

A genetic and phenotypic overlap between ADHD and dyslexia was also discussed. A well-studied risk gene for ADHD is SNCA (3rd), which encodes α-synuclein, a protein located primarily in the presynaptic vesicles, which has been suggested to play a role in the modulation of dopamine transporter function [93]. In ADHD, the concentration of dopamine and other neurotransmitters in the brain decreases, which weakens their inhibitory activities and leads to hyperactivity. Dopamine transporter is the target of psychostimulants for the treatment of ADHD and plays a key role in regulating the dopamine concentration in the synaptic cleft [94]. Dopaminergic neurons are mostly found in the ventral midbrain and transmit to the striatum and prefrontal cortex [95]. Executive function, learning, mood control, reward sensitivity, and other functions are all affected by this protein [96]. According to previous research, disruption of the dopamine signaling system is a significant event the neuropsychiatric etiology of ADHD symptoms and dyslexia [97].

Among these top genes, DRD2, DRD4, CNTNAP2 and GRIN2B (6th, 11th, 14th, 19th place in Table 1) are directly discussed in the literature in the context of the comorbidity of ADHD and dyslexia [98–101]. The DRD2 gene, located at 11Q23.1, encodes the D2 dopamine receptor, which is involved in dopaminergic synaptic transmission. The dopamine receptor is prevalent in dopamine-containing brain regions, most notably the neostriatum, olfactory nodule, substantia nigra, ventral tegmental area, and nucleus accumbens [102]. The DRD4 gene, which encodes the D4 dopamine receptor, is found in the p15.5 region of chromosome 11. The DRD4 receptor is found at the synaptic end of neurons and has a high affinity for dopamine and norepinephrine in the synaptic gap [103]. It is a catecholamine receptor that participates in the transmission of the DA neurotransmitter between neurons. The D2 and D4 are presynaptic dopamine receptors that regulate dopamine synthesis and release, as well as controlling motor behavior, drug misuse, hormone production, and antipsychotic targets in schizophrenia [104]. As an excitatory neurotransmitter in the brain, dopamine primarily governs a number of central nervous system activities. When dopamine levels in the brain are low, children’s brains lose their capacity to manage irrelevant inputs, causing them to selectively filter irrelevant stimuli and become unable to maintain focus, resulting in ADHD. Accordingly, the expression of DRD2 and DRD4 was observed to be up-regulated in ADHD [105]. DRD2 and DRD4 are established drug targets for the treatment of ADHD [106, 107] and a number of SNPs in these genes are associated with ADHD [108, 109]. Dopaminergic function is considered to be critical for the modulation of neural activity in the striato-thalamo-cortical circuit, which is involved in complex goal-directed or context-dependent changes in human speech and bird song output [110]. Moreover, the dopaminergic system also plays an important role in maintaining linguistic functions such as speech fluency and reading, and a number of genetic polymorphisms in this system have been identified as important risk factors for dyslexia. For instance, a dyslexia susceptibility locus (DYX7) has been found to be linked to the dopamine D4 receptor (DRD4) region on chromosome 11p15.5 in participants of European ancestry [111]. At the same time, an association between DRD2 and stuttering has been found in the Chinese population through high-density genotyping [112]. DRD2 and DRD4 mutations are linked to selective cognitive impairment of working memory and behavioral flexibility. Working memory is a type of temporary information processing and storage that aids in the coordination of many actions and tasks. People with dyslexia have been shown to have deficiencies not just in particular language abilities, but also in working memory. Thus, mutations in the DRD2 and DRD4 genes might potentially impair working memory in children with dyslexia. The DRD4 and DRD2 genes have been linked to ADHD and dyslexia comorbidity. However, given the vast disparities in the linguistic and genetic background of different populations, the association of the DRD4 and DRD2 genes and ADHD with dyslexia comorbidity warrants more investigation.

Other interesting genes are CNTNAP2 and GRIN2B, which ranked at the 14th and 19th places in Table 1, respectively. The CNTNAP2 gene, located at 7q35, encodes a neuronal adhesion molecule called presynaptic membrane extension protein, which is produced in the developing human cerebral cortex [113]. The presynaptic membrane extensor protein interacts with neuroligin in the postsynaptic membrane and is essential for the construction, differentiation, and information transmission function of synapses. Common variants in the CNTNAP2 gene have been linked to lower white and gray matter volume in the cerebellum, fusiform gyrus, occipital lobe, and frontal lobe, as well as structural connections across different brain areas, according to neuroimaging studies in healthy adults [114]. The CNTNAP2 gene has been linked to changes in regions related to language function and neural networks in the brain. Transgenic animal models imply that CNTNAP2 is required for appropriate brain development because it encodes glial adhesin in myelinated axons [115]. Neural progenitor cells from CNTNAP2 heterozygous patients exhibited a substantial decrease in neural migration, whereas hiPSC-derived neurons from carriers exhibited changes in the expression of genes associated with synaptic transmission and neuronal activity [101]. Some phenotypic traits are shared across species, such as hyperactivity, which is observed in both mice and zebrafish and is one of the most noticeable signs of ADHD [116]. These studies demonstrated that CNTNAP2 is a candidate gene contributing to ADHD and dyslexia comorbidity.

The NMDA receptors are a glutamate receptor family that is involved in the excitatory neurotransmission of Ca^2+^ signals in the central nervous system (CNS) [116]. They perform critical physiological functions in neuronal differentiation and migration, synaptic plasticity, and long-term hippocampal potential control. GRIN2B encodes the GluN2B subunit of the NMDA receptor, and is located on chromosome 12p13.1 [117]. High levels of GRIN2B protein expression are detected in the hippocampus and the medial prefrontal cortex, an important brain region for spatial learning and memory tasks [118]. In view of its importance in psychological cognition, GRIN2B mutations have been associated with a number of neurodevelopmental diseases, including ADHD and dyslexia.

### Biological processes underlying ADHD and dyslexia

Currently, there is increased interest among researchers in the effects of various pathological processes. The most relevant genes identified in this study were found to be involved in biological processes related to signal transduction, positive regulation of transcription from RNA polymerase II promoter, chemical synaptic transmission, response to drugs, ion transmembrane transport, nervous system development, cell adhesion, and neuronal migration. To further assess the biological processes potentially underlying ADHD/dyslexia, we evaluated the gene cluster by calculating the degree centrality (DC), total centrality (CC), and betweenness centrality (BC) using Cytoscape plugin Network Analyzer and R language. For a given network, each gene is characterized as a node and the interactions between the genes are known as edges. DC is the sum of edges linked to it, therefore, a high degree signifies the hub genes owning chief biological functions. BC shows the importance of a node by the number of small paths passing through each node. The BC of the node is computed as:

1 $${\rm{BC = \Sigma s}} \ne {\rm{n}} \ne {\rm{t(\sigma }}\,{\rm{st }}\left( {\rm{n}} \right){\rm{/\sigma }}\,{\rm{st)}}$$

Where, n is node, s and t are nodes in the network other than node n, σst denotes the number of shortest paths from s to t, and σst (n) is the number of shortest paths from s to t of node n. Whereas, CC is the number of connected pairs or edges between the nodes. It is defined as follows:

2 $${\rm{CC = }}{{\rm{2}}_{{\rm{en}}}}{\rm{/}}\left( {{{\rm{k}}_{\rm{n}}}\left[ {{{\rm{k}}_{\rm{n}}}{\rm{ - 1}}} \right]} \right)$$

Where, kn is the number of neighbors of node n and en is the number of connected pairs between all neighbors.

The value of BC and CC always lies between 0 and 1. Therefore, the decisive network was visualized centred on BC, CC and a node degree, and the hub genes were generated based on higher BC values, CC values, and the node degree with the cut off ≥ 0.005, ≥ 0.2 and > 40 respectively.

**Fig. 4:**
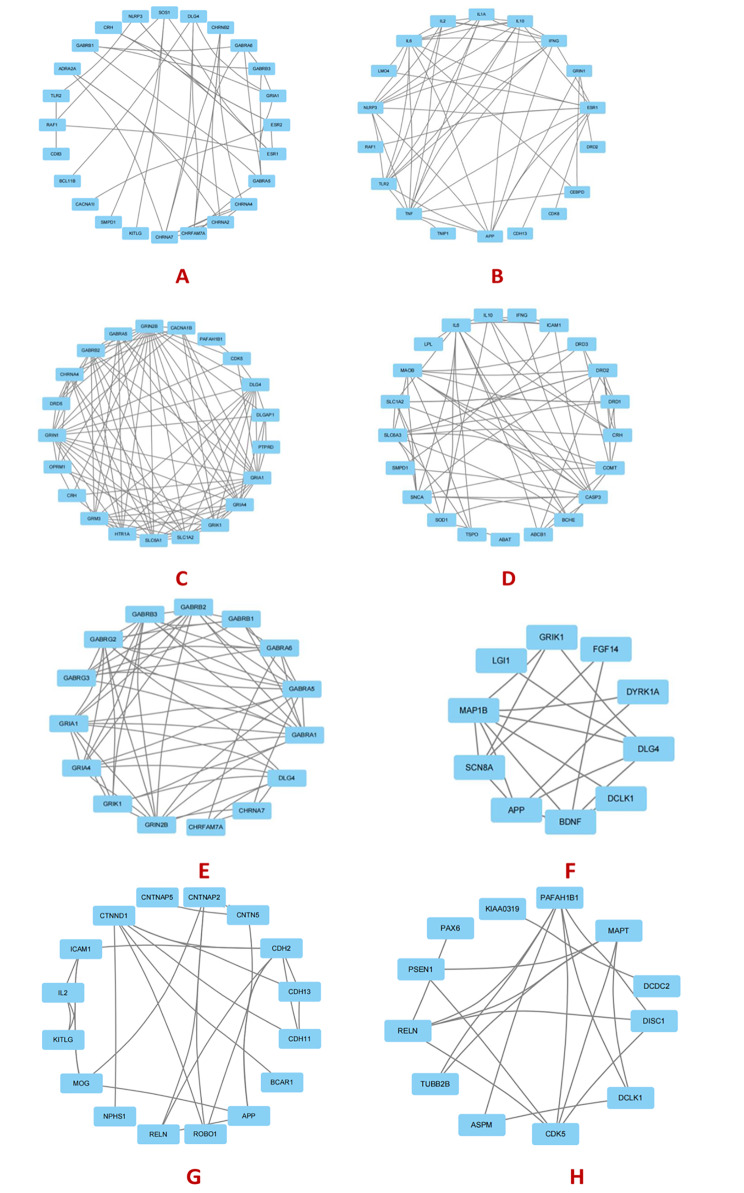
**(A)** signal transduction, **(B)** positive regulation of transcription from RNA polymerase II promoters, **(C)** chemical synaptic transmission, **(D)** response to drugs, **(E)** ion transmembrane transport, **(F)** nervous system development. The figure was plotted using Cytoscape. **(G)** cell adhesion, **(H)** neuronal migration. The figure was rendered using Cytoscape

In particular, GO:0007165 ~ signal transduction (Fig. 4A) plays the most important role among all biological processes for the complete network of ADHD/dyslexia. Signal transduction is mostly represented by the proteins GABRB3, GRIA1, GABRB1, CD83, CHRNA2, CHRNA4, CHRNA7, ELK3, CACNA1I, SMPD1, TSPO, NLRP3, FLNB, GTF2I, SH2B1, CHRNB2, BCL11B, GABRA6, GABRA5, ESR1, ADRA2A, ESR2, KITLG, FGF14, DLG4, CRH, and CHRFAM7A. The onset of dyslexia and ADHD is based on signal transduction, and is related to mechanisms involving neurotransmitters such as dopamine, adrenaline, and acetylcholine [119]. GO:0045944 ~ positive regulation of transcription from RNA polymerase II promoters (Fig. 4B) was represented by the proteins APP, CEBPD, TNF, ELK3, NEUROD6, NLRP3, DRD2, DRD3, IL10, AUTS2, GSX1, BCL11B, LMO4, PAX6, TBR1, ESR1, IL2, GRIN1, CDK8, PER1, IL1A, IL6, MEIS1, IFNG, TNIP1, TET3, CDH13, RAF1, and TLR2. The biological process of RNA polymerase II promoter transcription controls gene expression directly. A considerable number of studies in recent years have found links of RNA polymerase II promoter transcription with ADHD and dyslexia. The function of miRNAs in processes important to central nervous system development, such as cell proliferation and differentiation, synaptogenesis, synaptic plasticity, and apoptosis, might lead to the creation of novel biomarkers for the diagnosis and prognosis of ADHD and dyslexia [120]. GO:0007268 ~ chemical synaptic transmission (Fig. 4C) was represented by the proteins GRIA1, GABRB2, CHRNA4, GABRA5, CACNA1B, SLC1A2, HTR1A, GRIK1, OPRM1, SLC6A1, GRIN2B, GRIN1, GRM3, PTPRD, CDK5, DLG4, CRH, DLGAP1, GRIA4, DRD5, and PAFAH1B1. Chemical synaptic transmission regulates synaptosome-related protein transmission. Synaptic proteins, which are primarily synthesized in neurons, play a vital role in neurotransmitter release, attention management, behavioral inhibition, learning, planning and strategy formation, thinking and reasoning, as well as a variety of other higher cognitive tasks in the brain [121]. GO:0042493 ~ response to drugs (Fig. 4D) was represented by the proteins IL10, BCHE, ABCB1, MAOB, SLC1A2, LPL, ABAT, COMT, SLC6A3, ICAM1, SOD1, IL6, IFNG, CASP3, SMPD1, CRH, TSPO, DRD1, DRD2, DRD3, and SNCA. There is currently no pharmaceutical intervention for dyslexia. Several studies have been undertaken to treat dyslexia and ADHD due to their similar genetic origins. The medicine tomoxetine is used to treat the comorbidity of these two diseases. According to a study by Shaywitz et al. [122], tomoxetine improved both ADHD and reading symptoms in ADHD and dyslexia. Notably, the improvement of reading symptoms could not be explained by the improvement of ADHD symptoms. According to Shaywitz et al., tomoxetine can increase reading and decoding ability, as well as reading vocabulary capacity, in ADHD and dyslexic patients, and the improvement of reading performance of comorbid dyslexia was not directly caused by the treatment of ADHD symptoms [122]. This phenomenon might be explained by the biological mechanism of responses to drugs.

GO:0034220 ~ ion transmembrane transport (Fig. 4E) was represented by the proteins GABRB3, GRIA1, RYR1, GABRB2, GABRA1, GABRB1, GABRA6, GABRA5, CHRNA7, GRIK1, ATP2C2, GABRG3, GRIN2B, GABRG2, DLG4, CHRFAM7A, RAF1, and GRIA4. Ion transmembrane transport, especially Ca^2+^ transport, is significantly related to the comorbidity of ADHD and dyslexia. The ATP2C2 gene, for example, encodes an adenosine triphosphatase that transfers Ca^2+^ and Mn^2+^ to the Golgi lumen [123]. ATPases have recently been implicated in neurodevelopmental disorders [124]. Ca^2+^ regulates a number of neural functions, including neuronal migration, axon guidance, synaptic plasticity, memory, neuronal excitability, neurosecretion, and senescence [125, 126]. Excessive intracellular Mn^2+^ buildup is hazardous and can cause apoptosis as well as manganese poisoning, a neurological condition marked by tremors and convulsions [127]. Dysregulation of these cations has been linked to ADHD and dyslexia [124]. GO:0007399 ~ nervous system development (Fig. 4F) was represented by the proteins EPM2A, LGI1, APP, BDNF, DYRK1A, GRIK1, DCLK1, ROBO1, FGF14, MOBP, NEUROD6, DLG4, MAP1B, SMPD1, SCN8A, and TPP1. It has been shown that both ADHD and dyslexia are linked to abnormalities in critical genes involved in central nervous system development [128, 129]. GO:0007155 ~ cell adhesion (Fig. 4G) was represented by the proteins APP, CNTNAP2, MOG, CNTN5, CTNND1, IL2, ROBO1, ICAM1, KITLG, RELN, NPHS1, CDH2, CDH11, CDH13, CNTNAP5, and BCAR1. These genes encode cell adhesion molecules that are thought to have a function in brain development and were previously linked to ADHD and dyslexia [130]. GO:0001764 ~ neuronal migration (Fig. 4H) was represented by the genes CDKL5, DCDC2, PAX6, PSEN1, DCLK1, ASPM, TUBB2B, RELN, CDK5, MAPT, DISC1, KIAA0319, and PAFAH1B1. These genes encode proteins that can either directly or indirectly affect cytoskeletal microtubules and actin filaments, both of which are required for neuronal migration and axonal growth [131]. The pathogenic process underlying ADHD and dyslexia include aberrant cell migration [132].

## Conclusions

Computer reconstruction and analysis for gene networks opens up possibilities to the proposal of hypotheses concerning the molecular mechanisms underlying diseases. It has been also proved to be efficient in exploring the complicated correlations of comorbid diseases. The reconstructed ADHD/dyslexia gene network that depicts the latent molecular-genetic interactions of these two diseases contains 231 genes/proteins. With standard prioritization methods, and original criteria using the ADHD/dyslexia gene network structure, 20 candidate genes for genotyping and drug target search are raised. Genes OPRM1, CHRNA4, and SNCA had the highest priority. In addition, the highest ranking genes are engaged in biological processes associated with signal transduction, positive regulation on transcription from RNA polymerase II promoters, chemical synaptic transmission, response to drugs, ion transmembrane transport, nervous system development, cell adhesion, nd neuronal migration, which can all reasonably contribute to dyslexia and ADHD and their comorbidity. However, the roles of these genes remain unclear, worthy of attention in future experiments.

### Electronic supplementary material

Below is the link to the electronic supplementary material.

**Additional file 1: Table S1.** Lists of genes associated with asthma and Hypertension in ANDsystem and GeneCard

**Additional file 2: Table S2.** Results of gene ontology enrichment analysis

**Additional file 3: Table S3.** Criterion 1 results

**Additional file 4: Table S4.** Criterion 2 results

**Additional file 5: Table S5.** Criterion 3 results

**Additional file 6: Table S6.** Criteria 4 results

**Additional file 7: Table S7.** Criteria 5–6 results

**Additional file 8: Table S8.** Criterion 7 results

**Additional file 9: Table S9.** Criterion 8 results

**Additional file 10: Table S10.** Criteria 9–10 results

**Additional file 11: Table S11.** Total score results

## Data Availability

All data generated or analysed during this study are included in supplementary information files.
